# Distributed Fiberoptic Sensor for Simultaneous Humidity and Temperature Monitoring Based on Polyimide-Coated Optical Fibers

**DOI:** 10.3390/s19235279

**Published:** 2019-11-30

**Authors:** Pavol Stajanca, Konstantin Hicke, Katerina Krebber

**Affiliations:** Bundesanstalt für Materialforschung und–prüfung (BAM), Unter den Eichen 87, 12205 Berlin, Germany; konstantin.hicke@bam.de (K.H.); katerina.krebber@bam.de (K.K.)

**Keywords:** distributed humidity sensing, fiberoptic sensors, polyimide-coated optical fibers, optical frequency-domain reflectometry, dual sensing

## Abstract

Along temperature, humidity is one of the principal environmental factors that plays an important role in various application areas. Presented work investigates possibility of distributed fiberoptic humidity monitoring based on humidity-induced strain measurement in polyimide (PI)-coated optical fibers. Characterization of relative humidity (RH) and temperature response of four different commercial PI- and one acrylate-coated fiber was performed using optical backscattering reflectometry (OBR). The study addresses issues of temperature-humidity cross-sensitivity, fiber response stability, repeatability, and the influence of annealing. Acrylate-coated fiber exhibited rather unfavorable nonlinear RH response with strong temperature dependence, which makes it unsuitable for humidity sensing applications. On the other hand, humidity response of PI-coated fibers showed good linearity with fiber sensitivity slightly decreasing at rising temperatures. In the tested range, temperature sensitivity of the fibers remained humidity independent. Thermal annealing was shown to considerably improve and stabilize fiber RH response. Based on performed analysis, a 20 m sensor using the optimal PI-coated fibers was proposed and constructed. The sensor uses dual sensing fiber configuration for mutual decoupling and simultaneous measurement of temperature and RH variations. Using OBR, distributed dual temperature-RH monitoring with cm spatial resolution was demonstrated for the first time.

## 1. Introduction

Distributed fiberoptic sensors (DFOSs) have a unique ability to provide spatially continuous measurement over extended distances up to hundreds of kilometers [1]. This makes DFOSs especially attractive for monitoring of large civil, energy, or geotechnical structures such as bridges, tunnels, pipelines, power cables, dams, slopes, and others. Strain and temperature are the most common measurands for DFOSs, as these are the quantities to which the optical fibers are inherently sensitive. Nevertheless, a large effort is being continuously made to expand the DFOS applications also for monitoring of other measurands such as environmental factors or chemicals [2]. Humidity belongs to the principal environmental factors that is of importance in various fields, e.g., agriculture, civil engineering, structural health monitoring (SHM), or industrial process control. Distributed humidity/water monitoring is sought after for numerous applications including soil moisture measurement in agriculture [3], concrete condition monitoring in civil engineering [4], leak detection in sewage industry [5], corrosion prevention in pipeline industry [6], environmental monitoring for particle accelerator detectors [7], or SHM of large structures such as dykes or dams [8]. Despite notable applicational demand, available technical solutions for fiberoptic distributed humidity sensing (DHS) are rather scarce.

Conventional Raman- or Brillouin-based fiberoptic distributed temperature sensing with active (heated) sensing fiber was explored for a number of soil hydrologic monitoring applications [8,9,10,11]. Here, the moisture is monitored indirectly through measuring the temperature response of the buried fiber as the heat dissipation rate depends on the soil water content. The approach, however, suffers from serious practical and conceptual limitations associated with complex nature (e.g., spatial inhomogeneity and temporal instability) of fiber cable embedding conditions [12].

Another category of fiberoptic DHS relies on the measurement of humidity-induced attenuation (HIA). Various concepts have been explored to incur additional optical loss to the fiber upon interaction with water/moisture. A combination of water-swellable elements and smart fiber packaging was used to induce HIA via fiber bending losses [5,13]. Another approach relies on recoating part of the fiber with hydrophilic polymer that changes its refractive index as a function of ambient humidity, thus altering fiber guiding properties [14]. Inherent absorption of moisture into polymer matrix of certain plastic optical fibers and subsequent measurement of HIA from absorption on water molecules has been explored as well [15,16]. While the attenuation-based techniques can be relatively easily implemented using simple optical time-domain reflectometry (OTDR), their precision and (longitudinal) monitoring range is rather limited as HIA inherently deteriorates transmission properties of the sensing fiber.

At the moment, approaches based on measurement of humidity-induced strain (HIS) caused by a swelling of hygroscopic fiber coating materials seem to be the most promising and practical. While other coating materials, e.g., acrylates [6] or TiO_2_ [17], have been used for HIS-based fiberoptic humidity sensors as well, polyimide (PI) coatings are the most prevalent. The mechanism has been explored extensively in combination with point fiber Bragg grating (FBG) sensors [18,19,20,21]. Only more recently, Thomas and Hellevang investigated and demonstrated possibility of DHS using PI-coated fibers in combination with optical backscatter reflectometry (OBR) [22]. The same authors later produced a short piece of PI-coated fiber with varying coating thicknesses up to 1.3 mm and showed the possibility of increasing fiber HIS response [23]. Nevertheless, such optical fibers with thick PI coatings remain commercially unavailable as application of PI on the fiber relies on thermal curing and is limited to few µm layer thickness in a single step. Instead of interrogation with OBR, which is limited to relatively short distances (tens of meters), Neves et al. used phase-sensitive OTDR to extend the measurement range up to 10 km [7]. The increase in monitoring range, however, comes at the price of decreased spatial resolution. In addition to PI-coated fibers, acrylate-coated fibers in combination with OBR interrogation have been explored for DHS as well [6]. In our recent work, we showed that even tight-buffered optical fibers might be considered for certain qualitative water sensing applications, when maximization of fiber HIS response is the key requirement [24].

While previous works had a proof-of-concept character and focused on validation of HIS-based sensing mechanism, in this work, we address key practical aspects of HIS-based DHS and demonstrate distributed environmental sensing that allows decoupling of temperature, strain, and relative humidity (RH) effects. Temperature and RH response characterization of four different commercial PI- and one acrylate-coated fiber is performed. The analysis addresses issues of temperature-RH cross-sensitivity, fiber response stability, repeatability, and the influence of annealing with an aim of selecting the optimal sensing fiber (s). After thorough fiber characterization, short (20 m) sensing cable is developed that allows distributed environmental measurement with mutual discrimination of RH and temperature variations at simultaneous isolation from mechanical (strain) influences. Using OBR, HIS-based distributed dual temperature-RH environmental sensing with cm spatial resolution is demonstrated for the first time. The research is carried out in the context of power cable monitoring, targeting development of water ingress sensor for subsea cable joints. Nevertheless, the presented results remain relevant in the broader context of distributed humidity sensing and the developed sensor can be of interest in various other application areas.

## 2. Materials and Methods

Humidity and temperature response of five single-mode fibers (SMFs) was investigated in scope of this work. The fibers include four different commercial PI-coated fibers with different fiber-coating geometry and a standard acrylate-coated fiber as a reference. As the RH sensitivity of PI-coated fibers depends on the relative glass-to-coating area ratio [23], used fibers cover the typical range of fiber-coating geometry for PI-coated fibers available on the market. They also include two fibers with the same nominal fiber-coating characteristics from two different producers to test for cross-manufacturer variations in the fiber response. The overview of the tested fibers and their fiber-coating characteristics can be found in Table 1.

**Figure 1:**
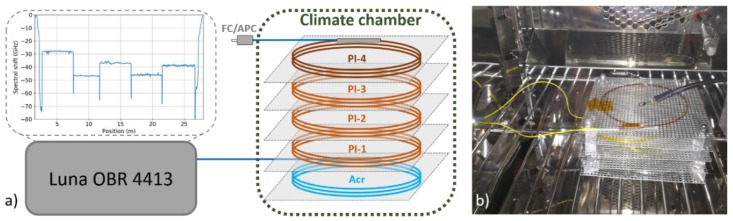
(**a**) Schematic illustration of experimental setup used for single-mode fibers (SMF) temperature-humidity characterization; (**b**) photo of the fiber sample holder inside of the climate chamber.

For the temperature-humidity characterization, roughly 5 m long segments were cut from each fiber and coiled into small spools with diameter of 6–8 cm. The individual spools were put into a compact shelf-type holder at separate levels and spliced together in order to allow simultaneous measurement of all fibers in series. The order of the fibers in the series as they were interrogated throughout the study is indicated in Figure 1a. Additional FC/APC pigtails were spliced at the beginning and the end of the fiber series for easy connection. The fiber holder was placed inside of a climate chamber while the pigtails were fed through a side opening outside to connect to a measurement device. A digital environmental sensor Ahlborn FHAD460L10 was placed inside the climate chamber close to the fibers for additional temperature and RH validation (Figure 1b). All the experiments were performed using Vötsch VCL 4003 climate chamber (CC) allowing simultaneous programmable control of temperature and relative humidity in 10–95 °C and 10%–95% range, respectively. Optical backscatter reflectometer OBR 4413 from Luna Technologies, based on swept-wavelength interferometry (sometimes also referred to as optical frequency-domain reflectometry) in the 1310 nm spectral region, was used to measure fiber temperature and RH response. Detailed explanation of OBR operating principle goes beyond the scope of this paper and can be found, for example, in ref. [25,26]. In short, the technique allows high-resolution distributed measurement of relative strain- or temperature-induced changes in fiber Rayleigh backscatter profile by cross-correlating local backscatter spectra from actual and reference measurements in the frequency domain. The measurement technique was selected due to its high sensitivity and spatial resolution, which is beneficial for the intended lab-scale characterization and demonstration. Nevertheless, the presented working principle and qualitative conclusions on fiber performance also remain valid if other DFOS techniques allowing distributed temperature and strain measurement are used.

For temperature characterization, an automated climate chamber program with 10 °C temperature steps from 10 °C to 50 °C and back with 2 h holding time at each temperature level was used. Fiber temperature response characterization was performed separately at 3 different constant relative humidity levels of 10%, 50%, and 90%. For humidity characterization, automated climate chamber program with 20% RH steps from 10% to 90% and back with 4 h holding time at each RH level was used. Fiber humidity response characterization was performed separately at 3 different constant temperature levels of 20 °C, 35 °C, and 50 °C. Throughout the CC program runtime, automated backscatter measurement with Luna OBR was taken each 5 or 10 min for temperature and humidity response characterization, respectively. All SMFs were interrogated simultaneously (in series) using a 15 nm scanning range and 5 cm spectral shift resolution. Relative spectral shift curves calculated by the device native software, as well as raw backscatter data, were saved for further post-processing. Entire temperature and humidity characterization series (2 × 3 measurement cycles) was firstly performed with pristine fibers and then repeated after the fibers had been annealed for 48 h at 50 °C and 90% RH. The upper operation temperature limit (50 °C) was chosen based on preliminary investigations, which indicated that prolonged operation at temperatures above 60 °C may deteriorate linearity of humidity response of some of the fibers (see Appendix A). Validation of the fiber performance outside tested temperature interval (20–50 °C) would require further investigations. Nevertheless, the considered temperature operational range sufficiently covers many practical scenarios including application targeted in our project, i.e., water ingress detection in subsea cable joints.

## 3. Results and Discussion

### 3.1. Fiber Characterization

**Figure 2:**
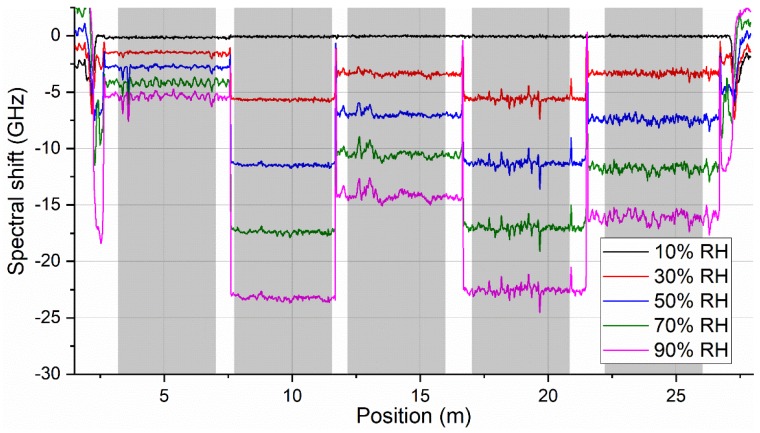
Illustration of measured optical backscattering reflectometry (OBR) spectral shift traces at different moments throughout a humidity characterization cycle (at 20 °C) corresponding to different relative humidity (RH) levels. Gray shaded areas indicate five sections for individual fibers that were considered for further data processing.

Figure 2 illustrates a typical evolution of measured OBR spectral shift traces at selected moments throughout a humidity cycle. Five distinct sections corresponding to individual fibers with different humidity response magnitudes are clearly visible. Areas at the edges of the graph correspond to the fiber pigtails leading out of the climate chamber.

Slight inhomogeneities of the spectral shift response within individual fiber sections are most likely caused by local restrictions in free movement (expansion) of the fibers within 5 m spools. This is hard to completely avoid for tight fiber spools with such small diameters. On the other hand, our preliminary studies showed that using fiber spools with considerably larger diameter led to even more severe inhomogeneities in the measured signal. These are associated with real spatial inhomogeneities of temperature and RH distribution within the climate chamber. Therefore, the selected fiber holder design targeted maximal compactness of the fiber assembly in order to minimize the impact of spatial temperature and RH inhomogeneities in the CC. Nevertheless, part of the signal fluctuations along the fiber sections might also come from coating inhomogeneities along the fiber.

**Figure 3:**
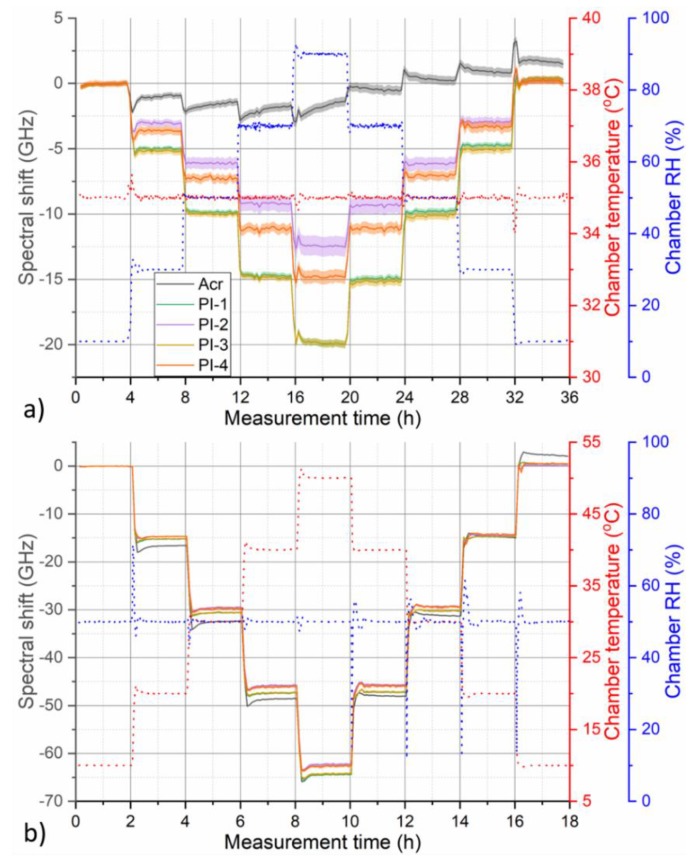
Temporal evolution of measured OBR spectral shift of pristine fibers for (**a**) humidity characterization cycle at 35 °C and (**b**) temperature characterization cycle at 50% RH. Shaded areas around the curves indicate standard deviation of the displayed fiber section-averaged values. Red and blue dotted lines show corresponding evolution of temperature and RH during the used climate chamber program, respectively.

To evaluate the evolution of fiber RH or temperature response throughout the characterization cycle, spectral shift values corresponding to the individual fiber sections were spatially averaged separately for each OBR trace recorded in a given measurement cycle. The extent (length) of averaged sections was kept the same for all five fibers and is indicated by shaded areas in Figure 2. Examples of spectral shift evolution graphs prepared in this way are presented in Figure 3. Figure 3a shows temporal evolution of OBR spectral shift of pristine fibers for humidity characterization cycle at 35 °C. Figure 3b shows temporal evolution of measured OBR spectral shift of pristine fibers for temperature characterization cycle at 50% RH. The Figure shows that while humidity response of the tested fibers may differ considerably, temperature response of all the fibers is very similar. The largest humidity response was exhibited by PI-1 and PI-3 fibers coming from different manufacturers but with the same fiber-coating geometry (125/155 µm) and almost identical humidity response. General observation on fiber RH response magnitude is in agreement with previous works that showed that RH sensitivity of PI-coated fibers correlates with relative glass-to-coating area ratio [23].

**Figure 4:**
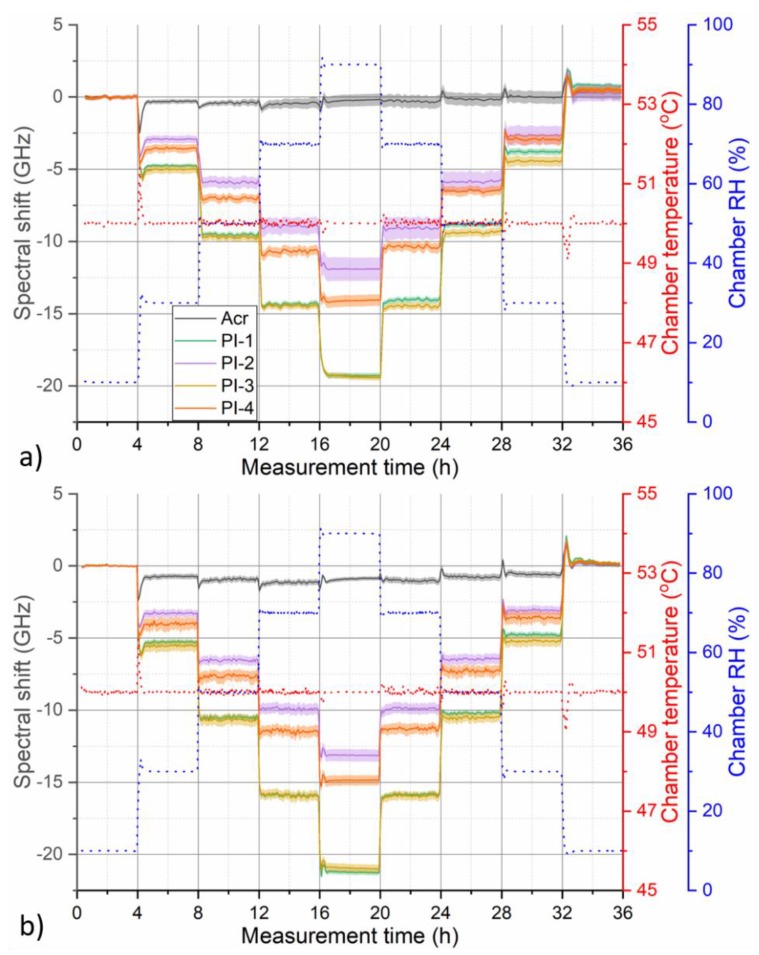
Temporal evolution of measured OBR spectral shift for tested fibers during humidity characterization cycle at 50 °C (**a**) before and (**b**) after annealing at 50 °C and 90% RH for 48 h. Shaded areas around the curves indicate standard deviation of the displayed fiber section-averaged values. Red and blue dotted lines show corresponding evolution of temperature and RH during the used climate chamber program, respectively.

While humidity response of PI-coated fibers followed stepwise changes of RH relatively nicely, response of acrylate-coated fiber exhibited more complicated behavior with significant hysteresis. This might be caused by thermally induced relaxation processes occurring in the coating polymer matrix, which leads to a permanent change of the coating characteristics and is typically associated with fiber longitudinal shrinkage. The effect is well known, for example, in the area of polymer optical fibers [27,28] and it can be partly mitigated by deliberate thermal pre-annealing of the fibers. Figure 4 compares measured spectral shift response during 50 °C humidity characterization cycle for pristine fibers and after they have been annealed at 50 °C and 90% RH for 48 h.

For humidity cycle at this temperature, slight hysteresis became visible even for pristine PI-coated fibers as the measured spectral shift curves did not return to their original zero value (Figure 4a). Annealing helped to minimize this hysteresis (Figure 4b). In addition, magnitude of RH response of PI-coated fibers increased slightly after annealing. Finally, the decreased uncertainty in displayed spectral shift curves indicates that annealing might also help to homogenize humidity response along the fiber. This is especially visible in the case of PI-2 and acrylate-coated fiber. Thermal pre-annealing might lead to relaxation of structural inhomogeneities in the coating polymer matrix that could be causing differences in humidity response along the fiber as observed in Figure 2. On the other hand, no significant improvement in the response of acrylate-coated fiber was observed after annealing. In fact, our investigations revealed that even after annealing, humidity response of the acrylate-coated fiber remains rather nonlinear with complex temporal character and strong temperature dependence.

**Figure 5:**
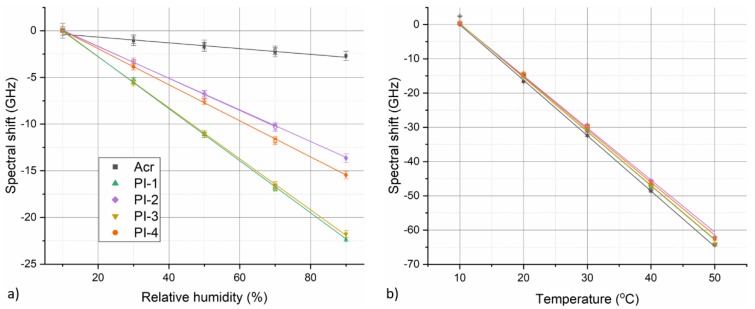
Fiber (**a**) humidity and (**b**) temperature response calibration graphs obtained for annealed fibers in the case of humidity cycle at 35 °C and temperature cycle at 50% RH, respectively. Data points from temperature or RH increase part of the respective cycles are shown as filled symbols, while data points from decrease parts of the cycles are shown as hollow symbols. Solid lines represent linear fits of the experimental data.

To further evaluate fiber temperature and relative humidity response, sensitivity of individual fibers was determined by plotting calculated section-averaged spectral shift values against corresponding temperature or RH step values. Fiber spectral shift values at individual RH or temperature steps were calculated as time-averaged values from the corresponding plateau regions of the temporal evolution curves (such as those depicted in Figure 3 or Figure 4). For humidity characterization measurements, this corresponds to roughly the last 1.5 h of each RH step. In the case of temperature characterization measurements, the averaged time interval corresponds roughly to last 0.5 h of each temperature step. Analogical approach was used for determining corresponding reference RH and temperature values from the climate chamber data. Figure 5 depicts examples of obtained calibration graphs for the case of humidity response at 35 °C and temperature response at 50% RH for annealed fibers. Fiber temperature $\alpha_{T}$ and relative humidity $\alpha_{RH}$ sensitivity coefficients under given conditions were determined using linear fit of the presented data. Datapoints from RH/temperature increase and decrease parts of the characterization cycles were fitted separately. As seen in Figure 5, the humidity responses of all PI-coated fibers exhibit very good linearity. Temperature response of the fibers is not ideally linear, rather the magnitude of spectral shift induced per °C change increases slightly with rising temperature. However, in the employed temperature range (10–50 °C), fiber temperature response can still be approximated by linear relation with good accuracy.

Fiber sensitivity coefficient for a given experiment was determined as an average of linear coefficients determined separately for increasing and decreasing part of the corresponding temperature or humidity cycle. Described evaluation approach was used for all temperature and RH characterization experiments at different conditions and before and after fiber annealing. Table 2 and Table 3 summarize all the determined humidity and temperature sensitivity coefficients, respectively. As mentioned earlier, temporal humidity response of acrylate-coated fiber at certain conditions remains complex, nonlinear, and exhibits notable hysteresis. Therefore, describing its response/sensitivity by a simple linear relation is rather unsuitable. Nevertheless, the evaluation is included for this fiber for the sake of completeness. In any case, results in Table 2 show that humidity response of acrylate-coated fiber suffered from strong temperature dependence, exhibiting dropping RH sensitivity at increasing temperatures. This, in combination with complicated nonlinear response, makes the acrylate-coated fiber unsuitable for any humidity sensing applications. At the same time, especially at low temperatures, humidity sensitivity of acrylate-coated fiber remains high enough to represent a notable cross-sensitivity issue in strain or temperature sensing applications; a fact that have remained largely overseen by many authors until now. For example, assuming $\alpha_{RH}$ = −0.087 ± 0.006 GHz/% RH (at 20 °C) and $\alpha_{T}$ = −1.64 ± 0.03 GHz/°C (at 50% RH) for a pristine acrylate-coated fiber, 10% variation in relative humidity would result in roughly 0.5 °C error in a temperature measurement if the humidity cross-sensitivity is not considered.

The trend of a slight humidity sensitivity decrease at rising temperatures was observed also for PI-coated fibers, both before and after annealing. While, for pristine fibers, the effect might have been caused by partial fiber annealing during the humidity cycle itself, the fact that this trend persists also after 48 h annealing at 50 °C reveals a true temperature dependence of fiber humidity sensitivity. This has not been identified in the previous studies on DHS with PI-coated fibers [22,23]. Discussion of the origin of this effect goes beyond the scope of this work, but it might be associated with softening of the polymer coating at higher temperatures and consequent decreased ability of HIS transfer to the fiber. To ultimately describe fiber RH sensitivity in the tested range of temperatures, mean $\bar{\alpha_{RH}}$ averaged from $\alpha_{RH}$ values determined for the three individual humidity cycles performed at different temperatures is used.

Decreased uncertainty degree of $\alpha_{RH}$ values determined at different temperatures for annealed fibers compared to pristine ones demonstrates the beneficial effect of pre-annealing by stabilizing fiber humidity response and removing hysteresis. As the displayed $\alpha_{RH}$ values were averaged from humidity increase and decrease part of the individual cycles, lower uncertainties indicate better agreement between humidity increase and decrease parts, hence lower hysteresis. At the same time, the temperature-averaged $\bar{\alpha_{RH}}$ of PI-fibers increases roughly by 10% for 125 µm fibers and 4% for 80 µm fiber after annealing. The sensitivity increase is beneficial for RH sensing applications as well.

Unlike humidity sensitivity, temperature sensitivity of all the fibers was fairly similar, around –1.6 GHz/°C, and was not influenced by the annealing (Table 3). This indicates that fiber temperature response is driven predominantly by the fiber itself (glass) and influence of the coating is rather negligible. Small humidity dependence of $\alpha_{T}$ was observed for the acrylate-coated fiber, while temperature sensitivity of all PI-coated fibers remained stable (within measurement error limits) under all tested environmental conditions. Nevertheless, mean $\bar{\alpha_{T}}$ averaged from $\alpha_{T}$ values determined for the three individual temperature cycles performed at different RH levels was used to ultimately characterize fiber temperature sensitivity in analogy to $\bar{\alpha_{RH}}$ values.

### 3.2. Distributed Sensing

The main motivation of the performed characterization study is selection of the most suitable fiber and realization of DHS. For the majority of practical implementations of HIS-based distributed humidity sensing, measurement of HIS alone would not be sufficient. As optical fibers are inherently sensitive to strain and temperature as well, the sensor needs to be able to discriminate between the effects coming from different measurands. Strain cross-sensitivity might be relatively straightforwardly minimized by mechanical isolation of sensing fiber, e.g., by placing it in a protective conduit. On the other hand, mitigation of temperature cross-sensitivity will require employment of a dual-sensing scheme to decouple the temperature- and humidity-induced signals. Ideally, additional humidity-insensitive sensing fiber would be used to compensate for temperature effects. Using metal-coated fibers or hermetically isolating the fiber from the environment (e.g., by placing it in a metal capillary) might be relevant strategies for obtaining reference temperature measurement free of impacts of humidity variation. However, we deem these approaches rather unpractical due to price- and/or handling-related factors. Here, two parallel sensing fibers with different temperature and RH sensitivities were used simultaneously. Spectral shift signal ν measured by the individual fibers as a result of temperature ∆T or relative humidity ∆RH change can be expressed in matrix form as

(1) $$\left(\begin{array}{c} \nu^{\left(1\right)}\\ \nu^{\left(2\right)} \end{array}\right)=\left(\begin{array}{cc} \alpha_{T}^{\left(1\right)} & \alpha_{RH}^{\left(1\right)}\\ \alpha_{T}^{\left(2\right)} & \alpha_{RH}^{\left(2\right)} \end{array}\right)\left(\begin{array}{c} ∆T\\ ∆RH \end{array}\right)$$

where upper index denotes one of the two sensing fibers. Since both sensing fibers experience the same environmental conditions, Expression (1) can be used to decouple the temperature and RH changes as

(2) $$∆T=\frac{\alpha_{RH}^{\left(1\right)}\nu^{\left(2\right)}-\alpha_{RH}^{\left(2\right)}\nu^{\left(1\right)}}{\alpha_{T}^{\left(2\right)}\alpha_{RH}^{\left(1\right)}-\alpha_{T}^{\left(1\right)}\alpha_{RH}^{\left(2\right)}}$$

and

(3) $$∆RH=\frac{\alpha_{T}^{\left(2\right)}\nu^{\left(1\right)}-\alpha_{T}^{\left(1\right)}\nu^{\left(2\right)}}{\alpha_{T}^{\left(2\right)}\alpha_{RH}^{\left(1\right)}-\alpha_{T}^{\left(1\right)}\alpha_{RH}^{\left(2\right)}}.$$

Effectiveness of the decoupling increases with increasing dissimilarity of sensitivity ratio $\alpha_{T}/\alpha_{RH}$ between the two fibers. Since all tested fibers have similar $\alpha_{T}$, using two fibers exhibiting highest contrast of $\alpha_{RH}$ would be optimal for the sensor operation. Very similar maximal $\bar{\alpha_{RH}}$ values of −0.28 ± 0.01 GHz/% and −0.274 ± 0.009 GHz/% were exhibited by PI-1 and PI-3 fiber, respectively. In the frame of this work, PI-1 was selected as the first sensing fiber, although, PI-3 would be an equally suitable candidate. While acrylate-coated fiber exhibited the lowest humidity sensitivity, discussed issues (nonlinearity, hysteresis, and strong temperature dependence of its RH response) make the fiber unsuitable for sensing applications. Therefore, PI-2 fiber with $\bar{\alpha_{RH}}$ of −0.170 ± 0.005 GHz/% was selected as the second sensing fiber.

**Figure 6:**
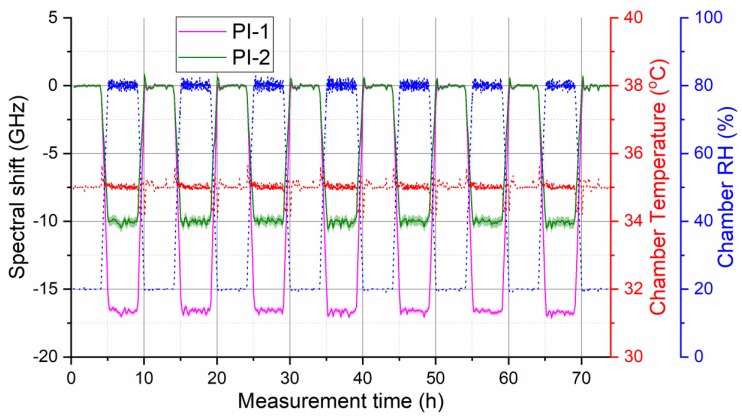
Measured humidity response of the two selected annealed polyimide (PI)-coated fibers during humidity cycling experiment demonstrating good stability and reproducibility of their response. Shaded areas around the curves indicate standard deviation of the displayed fiber section-averaged values. Red and blue dotted lines show corresponding evolution of temperature and RH during the used climate chamber program, respectively.

Stability and reproducibility of fiber humidity response is important for the reliable sensor operation. Figure 6 depicts the evolution of humidity-induced OBR spectral shift for the both selected fibers during climate chamber program with seven cyclic RH changes between 20% and 80% RH at a constant temperature of 35 °C measured over 74 h. The measurement confirms good stability and reproducibility of humidity response for both selected fibers after annealing.

**Figure 7:**
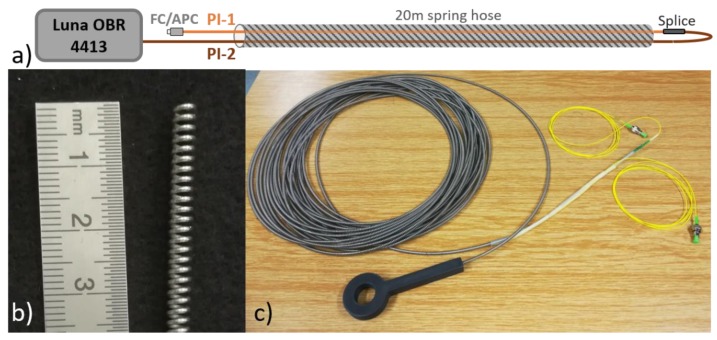
(**a**) Schematic depiction of prepared sensing cable; (**b**) detail photo of the metal spring hose used for mechanical protection and isolation of the sensing fibers; (**c**) photo of prepared sensing cable including protective 3D-printed housing for the end-splice and FC/APC pigtails for connection.

To demonstrate the distributed environmental sensing with simultaneous measurement of relative humidity and temperature, a small-scale sensing cable was prepared using the two selected PI-coated fibers (Figure 7a). The fibers were inserted in a 20-m-long metal spring hose (Figure 7b). The spring has an outer diameter of 4 mm, 1.6 mm helix pitch, and is made of a 0.8 mm thick stainless-steel wire. The spring hose provides mechanical protection for the fibers and minimizes undesired impact of strain cross-sensitivity while allowing the sensing fibers to interact with the surrounding environment. On one end of the hose, FC/APC pigtails were spliced to the PI-coated fibers, while the fibers were spliced together at the other end of the hose. The splice between the PI-coated fibers was placed in a protective 3D-printed plastic housing glued to the end of the spring hose. Figure 7a,c shows a schematic view and a photo of the prepared sensing cable, respectively. In accordance to performed sensitivity characterization and analysis, the cable was pre-annealed at 50 °C and 90% RH for three days. Fiber sensitivity coefficients were verified for the cable using the same approach as previously for the plain fibers, however, limited only to humidity cycle at 35 °C and temperature cycle at 50% RH. Temperature sensitivity coefficients of −1.62 ± 0.03 GHz/°C and -1.56 ± 0.03 GHz/°C were determined for PI-1 and PI-2 fibers, respectively. Humidity sensitivity coefficients of the fibers were −0.281 ± 0.001 GHz/% and −0.174 ± 0.001 GHz/%, respectively. All these values agree well with previously determined coefficients. For the purposes of evaluation of data measured with the prepared cable, temperature-averaged sensitivity values $\bar{\alpha_{RH}}$ and $\bar{\alpha_{T}}$ from Table 2 and Table 3 were used for the both sensing fibers. Generally, if high-precision humidity sensing is desired, temperature dependence of $\alpha_{RH}$ needs to be considered, which can complicate data evaluation notably. If courser measurement is sufficient, the temperature dependence of $\alpha_{RH}\;$ can be neglected for simplicity and fiber characterization by a single $\bar{\alpha_{RH}}$ value is sufficient. For the temperature range considered in this work (20–50 °C), the error associated with this simplification should stay within ± 5% of the measured RH change.

**Figure 8:**
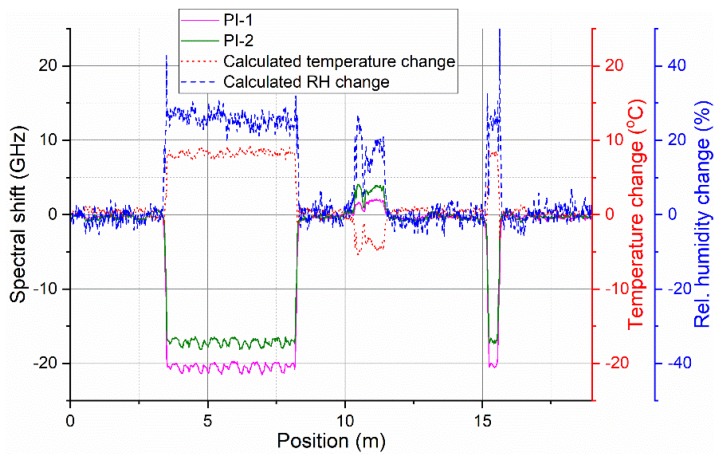
Example of overlaid spectral shift traces from both sensing fibers and corresponding calculated relative temperature and RH change.

To test possibility of distributed environmental sensing, two sections of the prepared sensing cable were put into the climate chamber. A roughly 5-m-long section of the cable (3–8 m from the connectorized end of the cable) was coiled inside of the climate chamber. An additional 0.5-m-long section (roughly at 15 m from the connectorized end of the cable) was fed directly (straight) through the side openings of the chamber. The rest of the sensing cable was placed freely around the room. Spectral shift evolution from the entire cable was recorded during a 54-h climate chamber program with simultaneous change of temperature and RH. The program consisted of two temperature ramp cycles between 20 °C and 40 °C and a simultaneous step-wise RH cycle from 10% to 90% and back in 20% steps. Automated Luna OBR measurement every 20 min, with the same settings as for general fiber characterization, was used. To decouple the effects of temperature and RH, separate spectral shift traces from the two sensing fibers were overlaid in the postprocessing and relative change of temperature and RH were calculated using Equations (2) and (3), respectively, at each point along the sensing fiber. The process is illustrated in Figure 8.

**Figure 9:**
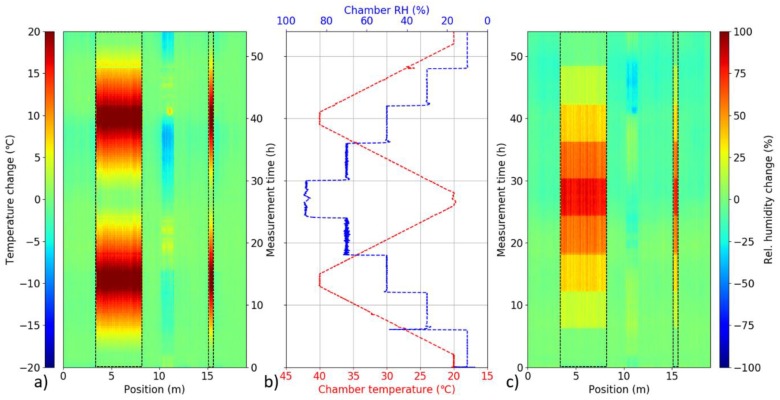
Measured temporal evolution of relative (**a**) temperature and (**c**) RH change along the sensing cable during the temperature-humidity climate chamber cycle (**b**). Cable segments placed inside of the climate chamber are highlighted by dashed rectangles.

Figure 9 depicts the overall 3D maps of calculated spatial-temporal distribution of relative temperature and RH change along the sensing cable during the temperature-humidity cycle. Both longer and shorter sensing cable segment inside of the climate chamber are clearly identifiable as they exhibit noticeable temperature (Figure 9a) and RH (Figure 9c) change that appears to follow the climate chamber program (Figure 9b). Temperature ramps as well as step-wise relative humidity cycle is clearly visible for the calculated decoupled 3D temperature and RH maps. The signal from the rest of the sensing cable outside of the climate chamber stayed relatively stable, indicating stable environmental conditions in the room. A notable exception is the section roughly at 10–11 m. This corresponds to the cable section that was placed deliberately directly into an open window and the observed temperature and RH changes reflect day cycle of outside environmental conditions.

**Figure 10:**
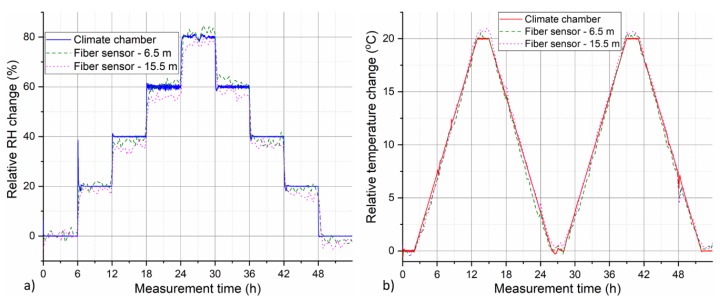
Comparison of experimentally measured and reference climate chamber (**a**) RH and (**b**) temperature evolution for the two selected positions along the sensing cable.

Figure 10 compares measured relative temperature and RH change from two selected positions along the cable with the reference values from the climate chamber. The first selected position (circa at 6.5 m) represents the longer coiled 5 m cable segment and the other one (circa at 15.5 m) represents shorter straight cable segment. Not only excellent qualitative, but also reasonable quantitative, agreement between the measured and reference curves is observed. Determined relative RH change stays within ± 5% RH of the reference value, while determined relative temperature change generally stays within ± 1 °C of the reference climate chamber value. Several different factors have an influence on accuracy of the presented sensor in our measurements. These include spatial inhomogeneities of temperature and RH distribution inside of the climate chamber, accuracy of the used OBR measurement device, uncertainty of determined fiber sensitivity values, or temperature dependence of $\alpha_{RH}$ that was neglected for the purposes of this work. Despite these limitations, we demonstrated the first HIS-based distributed humidity sensing with simultaneous discrimination of temperature cross-sensitivity. Spatial resolution of the sensor is defined by the used measurement device. In our case, it was 5 cm, as determined by the used spectral shift resolution of OBR device, which can be decreased down to sub-centimeter range. OBR was chosen for this study due to its sensitivity and high spatial resolution. On the other hand, its monitoring range is limited to a few tens of meters. Nevertheless, the presented sensing approach should be readily employable with other DFOS techniques such as phase-sensitive OTDR that can provide HIS measurement over much longer distances.

## 4. Conclusions

In this work, development and demonstration of distributed fiberoptic sensor for simultaneous temperature and relative humidity monitoring was reported. The investigated humidity measurement principle relies on humidity-induced strain of polyimide-coated optical fibers. The sensor uses dual sensing fiber configuration for mutual decoupling and simultaneous measurement of temperature and RH variations. Optical backscatter reflectometry was used as an interrogation technique throughout this work. In the first step, thorough characterization of RH and temperature response of four different commercial PI- and one acrylate-coated fiber was performed with the aim of analyzing their sensing performance and selecting most suitable candidates. Humidity response of acrylate-coated fiber was shown to have unfavorable nonlinear nature and strong temperature dependence with increasing RH sensitivity at lower temperatures. While this makes acrylate-coated fiber rather unsuitable for RH sensing tasks, its RH sensitivity still should be considered for high precision temperature or strain sensing applications, especially at low temperatures. In contrast to acrylate-coated fiber, all tested PI-coated fibers exhibited RH response with good linearity and superior stability. A similar trend of slight RH sensitivity increase at decreasing temperatures was observed for all PI-coated fibers. Depending on the application precision requirements and expected temperature operation range, the effect might need to be considered. For the temperature range considered in this work (20–50 °C), the effect may be neglected while keeping the introduced measurement error below 5% of measured RH change. In the tested range, temperature sensitivity of the PI-coated fibers remained humidity independent. Thermal annealing was shown to considerably improve RH response of PI-coated fibers in terms of minimizing the hysteresis, homogenizing the response along the fiber and increasing RH sensitivity. Based on performed analysis, the two most suitable fibers with similar temperature but different RH sensitivity were selected for development of targeted sensor. Sample 20-m sensor cable using the selected PI-coated fibers was proposed and constructed. The cable used metal spring hose to provide mechanical insulation for the sensing fibers while allowing them to interact with the environment. Using OBR and dual fiber configuration, distributed simultaneous temperature and RH measurement with cm spatial resolution was demonstrated for the first time. Although OBR was used for all the measurements in this work, the presented sensing approach should be readily employable with other DFOS techniques such as phase-sensitive OTDR. Similarly, all qualitative conclusions on fiber performance remain generally valid.

## Figures and Tables

**Table 1:** List of investigated single-mode fibers with their selected fiber-coating characteristics.

Fiber Code	Fiber Diameter	Coating Diameter	Coating Material
Acr	125.0 ± 0.7 µm	245 ± 5 µm	Acrylate
PI-1	125 ± 2 µm	155 ± 5 µm	Polyimide
PI-2	125 ± 1 µm	145 ± 3 µm	Polyimide
PI-3	125 ± 1 µm	155 ± 5 µm	Polyimide
PI-4	80 ± 2 µm	102 ± 5 µm	Polyimide

**Table 2:** Overview of determined fiber relative humidity sensitivity coefficients $\alpha_{RH}$ (in GHz/% RH).

Experiment	Acr	PI-1	PI-2	PI-3	PI-4
Pristine (20 °C)	−0.087 ± 0.006	−0.254 ± 0.003	−0.158 ± 0.003	−0.252 ± 0.003	−0.187 ± 0.005
Pristine (35 °C)	−0.031 ± 0.009	−0.251 ± 0.005	−0.155 ± 0.002	−0.251 ± 0.003	−0.186 ± 0.002
Pristine (50 °C)	−0.007 ± 0.002	−0.245 ± 0.008	−0.151 ± 0.004	−0.246 ± 0.005	−0.179 ± 0.003
**Average** $\bar{\alpha_{RH}}$ **Pristine**	**−0.04 ± 0.04**	**−0.250 ± 0.005**	**−0.155 ± 0.004**	**−0.249 ± 0.004**	**−0.184 ± 0.005**
Annealed (20 °C)	−0.070 ± 0.004	−0.290 ± 0.003	−0.174 ± 0.002	−0.283 ± 0.003	−0.193 ± 0.008
Annealed (35 °C)	−0.032 ± 0.002	−0.280 ± 0.002	−0.171 ± 0.001	−0.275 ± 0.002	−0.195 ± 0.002
Annealed (50 °C)	−0.013 ± 0.003	−0.266 ± 0.002	−0.165 ± 0.001	−0.265 ± 0.002	−0.188 ± 0.001
**Average** $\bar{\alpha_{RH}}$ **Annealed**	**−0.04 ± 0.03**	**−0.28 ± 0.01**	**−0.170 ± 0.005**	**−0.274 ± 0.009**	**−0.192 ± 0.004**

**Table 3:** Overview of determined fiber temperature sensitivity coefficients $\alpha_{T}$ (in GHz/°C).

Experiment	Acr	PI-1	PI-2	PI-3	PI-4
Pristine (10%)	−1.71 ± 0.01	−1.63 ± 0.03	−1.55 ± 0.03	−1.61 ± 0.03	−1.55 ± 0.02
Pristine (50%)	−1.64 ± 0.03	−1.6 ± 0.02	−1.53 ± 0.03	−1.58 ± 0.03	−1.55 ± 0.02
Pristine (90%)	−1.57 ± 0.06	−1.59 ± 0.01	−1.54 ± 0.02	−1.59 ± 0.01	−1.54 ± 0.02
**Average (** $\bar{\alpha_{T}}$ **) Pristine**	**−1.64 ± 0.07**	**−1.61 ± 0.02**	**−1.54 ± 0.02**	**−1.60 ± 0.02**	**−1.55 ± 0.02**
Annealed (10%)	−1.74 ± 0.03	−1.64 ± 0.03	−1.57 ± 0.03	−1.62 ± 0.02	−1.56 ± 0.02
Annealed (50%)	−1.65 ± 0.04	−1.59 ± 0.02	−1.53 ± 0.02	−1.59 ± 0.03	−1.55 ± 0.03
Annealed (90%)	−1.57 ± 0.06	−1.59 ± 0.02	−1.54 ± 0.02	−1.58 ± 0.02	−1.55 ± 0.02
**Average (** $\bar{\alpha_{T}}$ **) Annealed**	**−1.65 ± 0.08**	**−1.61 ± 0.03**	**−1.55 ± 0.02**	**−1.6 ± 0.02**	**−1.55 ± 0.02**

**Figure A1:**
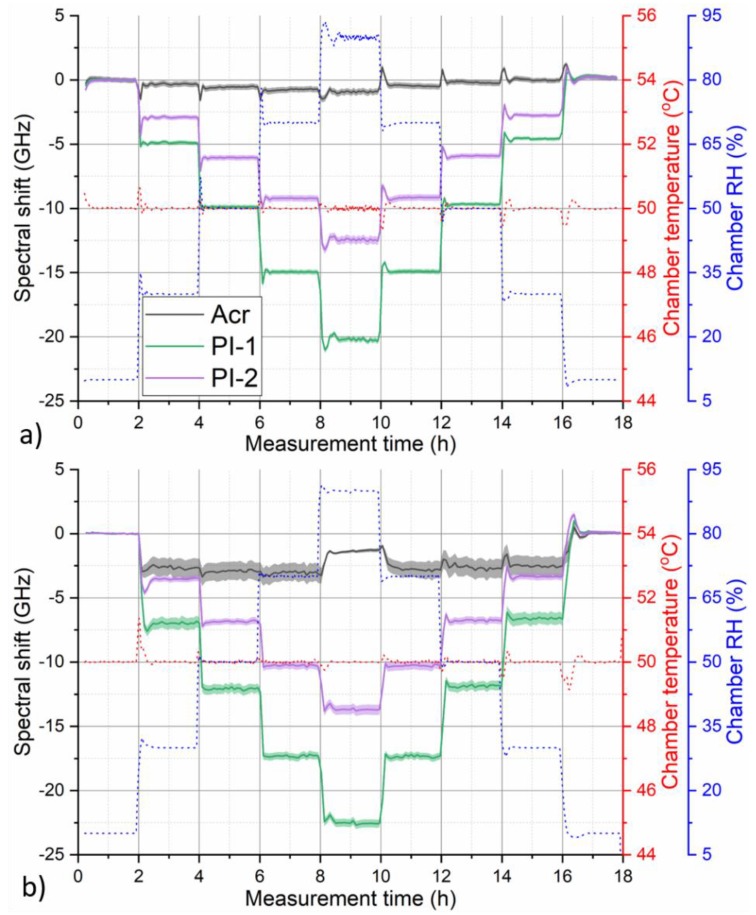
Temporal evolution of measured OBR spectral shift for selected fibers during humidity characterization cycle at 50 °C (**a**) before and (**b**) after annealing at 60 °C and 90% RH for 72 h. Shaded areas around the curves indicate standard deviation of the displayed fiber section-averaged values. Red and blue dotted lines show corresponding evolution of temperature and RH during the used climate chamber program, respectively.

**Figure A2:**
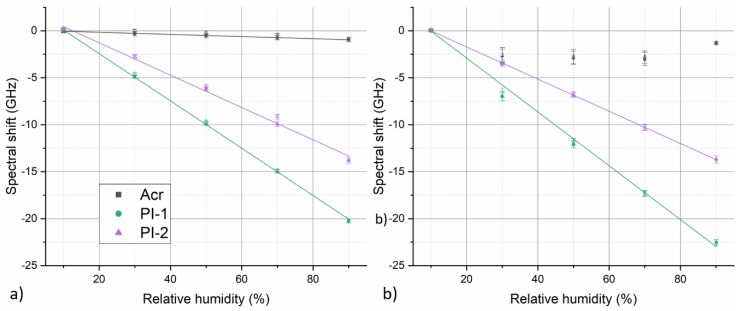
Humidity response calibration graphs for selected fibers (**a**) before and (**b**) after annealing in the case of humidity cycle at 50 °C. Data points from RH increase part of the cycle are shown as filled symbols, while data points from decrease parts of the cycle are shown as hollow symbols. Solid lines represent linear fits of the experimental data.

## Appendix A

Temperature stability of fiber humidity response at temperatures higher than 50 °C was investigated in preliminary trials. In these trials, also fiber annealing at 60 °C and 90% RH for 72 h was tested. In analogy to Figure 4, Figure A1 compares measured spectral shift response during 50 °C humidity characterization cycle for selected fibers before and after annealing. For the sake of clarity, only results for acrylate and two PI-coated fibers used in the presented sensing cable (PI-1 and PI-2) are displayed. Figure A2 depicts corresponding humidity response calibration graphs.

The results show that the humidity response of the acrylate fiber after the annealing becomes nonlinear and non-monotonic, thus completely unsuitable for RH sensing applications. Annealing at a higher temperature (compared to 50 °C presented in the manuscript) still generally helps to remove the hysteresis and increase sensitivity of RH response of PI-coated fibers. However, for some fibers, it may lead to notable deterioration of RH response linearity. This is illustrated in Figure A2b, where degraded linearity of PI-1 RH response is clearly visible, while the response of PI-2 remains almost perfectly linear. The measurement was repeated several times to verify the results with the same outcome.

While some of the tested PI-fibers may still offer stable RH response at temperatures above 50 °C, for the sake of completeness of the fiber characterization, testing temperature range was set to 20–50 °C where all of the tested PI-fibers could still provide reliable performance. Rigorous validation of the fiber performance outside tested temperature interval would require further investigations. Nevertheless, considered temperature operational range sufficiently covers many practical scenarios including application targeted in this work, i.e., water ingress detection in subsea cable joints.
